# Exploring the biological hallmarks of cancer of unknown primary: where do we stand today?

**DOI:** 10.1038/s41416-019-0723-z

**Published:** 2020-02-11

**Authors:** Elie Rassy, Tarek Assi, Nicholas Pavlidis

**Affiliations:** 1 Department of Medical Oncology, Institut Gustave Roussy, Villejuif, France; 2 Faculty of Medicine, Department of Oncology, Saint Joseph University, Beirut, Lebanon; 3 University of Ioannina, Ioannina, Greece

**Keywords:** Cancer of unknown primary, Molecular biology

## Abstract

Cancer of unknown primary (CUP) affects a small percentage of the general population. Nonetheless, a substantial number of these patients have a poor prognosis and consequently succumb to their illness within a year of diagnosis. The natural history of CUP is characterised by early metastasis from the unknown primary site, aggressive course and resistance to conventional chemotherapy. Unfortunately, the processes by which this orphan disease originates and progresses have not been fully elucidated and its biology remain unclear. Despite the conceptual progress in genetic and molecular profiling made over the past decade, recognition of the genetic and molecular abnormalities involved in CUP, as well as the identification of the tissue of origin remain unresolved issues. This review will outline the biology of CUP by exploring the hallmarks of cancer in order to rationalise the complexities of this enigmatic syndrome. This approach will help the reader to understand where research efforts currently stand and the pitfalls of this quest.

## Background

Cancer of unknown primary (CUP) is a term applied to a heterogeneous group of metastatic tumours, the primary sites for which cannot be identified at the time of diagnosis, despite extensive investigations.^1^ It is categorised into four major histological types that include adenocarcinoma of good-to-moderate differentiation (50%), followed by poorly undifferentiated adenocarcinomas (30%), squamous cell carcinoma (15%) and undifferentiated neoplasms (5%).^1^ The current standard approach to CUP relies on classifying patients into two prognostic subsets according to their clinicopathological characteristics: the minority of patients (15–20%) have a favourable prognosis and achieve a median survival of 10–16 months and long-term disease control in 30–60% of cases; by contrast, the majority of patients have an unfavourable prognosis with a dismal survival of 3–6 months despite management with a variety of chemotherapeutic combinations.^2^

The available literature reports a higher percentage of treatment receipt among patients with known primary than those with CUP (77.2% versus 51.1%), which supports a better survival for patients with metastatic cancer of a known primary site compared with patients with unknown primaries (11.9 versus 1.9 months).^3^ This finding constitutes the backdrop for the rationale to identify the primary tumour to provide better treatment outcomes. However, in this regard, the evaluation of common serum tumour markers has no diagnostic, prognostic or predictive value for CUP, with epithelial serum tumour markers (such as carcinoembryonic antigen, CA15-3, CA19-9 and CA-125) commonly showing non-specific overexpression.^4^ PET/CT scans by using fluorodeoxyglucose offer greater promise, with a sensitivity of 87% and specificity of 71%, indicating that few false-negative results occur, which is an important feature in the initial stages of the management process.^5^ Molecular gene profiling has yielded an identification rate of 77–94% using second-generation microRNA-based assays, gene expression profiling-based microarray tests or quantitative-PCR low-density arrays.^6^

As these radiological and molecular advances yield a higher identification rate than the standard approach by using tumour biomarkers, they therefore challenge the concept of unknown primary.^6–8^ Indeed, the incidence of CUP has decreased from around 3–5% in the 1990s to 1–2% in the current era.^9^ Unfortunately, however, the advances in diagnostics have not translated into a survival benefit, as no differences in outcome were reported between empirical and molecularly guided treatments.^10–12^ In this paper, we review the literature documenting advances in the molecular profiling of CUP in an attempt to disentangle its biology.

## The enigmatic entity of CUP

CUP can be viewed as an enigmatic cancer, as the accuracy of the diagnosis and the efficacy of the treatment are questioned.^2,13^ The standard approach to diagnosing patients with CUP includes a thorough history and examination, basic blood and biochemical tests and CT scans of the thorax, abdomen and pelvis in order to try to identify the source of the primary tumour; if the primary tumour cannot be located, then the cancer can be confirmed as being of unknown primary origin.^1^ These investigations constitute the bare minimum of tests recommended, with additional tests being suggested according to the clinical presentation, which highly depends on the experience of the treating oncologist.^14,15^

Subsequently, however, the diagnosis of CUP might be called into question in many situations. The first situation includes patients for whom suboptimal investigations have led to a false or premature diagnosis of CUP.^16,17^ A second scenario occurs in 10–20% of patients in whom the primary tumour is revealed during the disease course after the diagnosis of CUP.^18^ A third scenario applies in patients with CUP who undergo gene expression profiling to identify a primary tumour of origin (Table 1): the assumed circulating tumour cells probably undergo extensive immunoediting, which raises doubts over the accuracy of the diagnosis because the prediction of the gene expression profiling cannot be fully retained.^19^ In general, however, the majority of patients have a correct diagnosis of CUP with occasionally suggestive features of a putative primary tumour. Nevertheless, CUP seems to display a distinct natural history that differs from that of a metastatic tumour with a known primary origin.^2,20,21^ Moreover, CUP has been hypothesised to possess a genetic signature that is specific for its primary site and a second genetic signature that is primary-independent, pro-metastatic and possibly CUP-specific, which differentiates the known and unknown primary tumours.^22^

**Table 1:** Overview of the CUP studies evaluating the accuracy of tissue-of-origin prediction with profiling and classical diagnostic methods.

Study	*N*	Type of tissue analyzed/analyte	Profiling diagnostic methods	Tissue-of- origin prediction	Classical diagnostic methods
Tothill et al.^90^	13	FF/FFPE/RNA	GEP (microarray)	84%	Clinicopathological features
Horlings et al.^91^	38	FFPE/RNA	GEP (microarray)	64–94%	Clinicopathological features
Bridgewater et al.^92^	21	FFPE/RNA	GEP (microarray)	67%	Clinicopathological features
Varadhachary et al.^93^	104	FFPE/RNA	qRT-PCR	61%	Clinicopathological features
Van Laar et al.^94^	13	FF/FFPE/RNA	GEP (microarray)	92%	Clinical features
Monzon et al.^95^	21	FF/RNA	GEP (microarray)	76%	Clinicopathological features
Ferracin et al.^96^	16	FFPE/microRNA	47-miRNA signatures	75%	Conventional histology
Varadhachary et al.*^97^	87	FFPE/microRNA	48-miRNA signatures	84%	Clinicopathological features
Thompson et al.^98^	171	FFPE/NA	MTP (biotheranostics)	84%	Conventional histology
Fernandez et al.^99^	42	FF/DNA	Methylation array (1505 CpGs)	78%	Clinicopathological features
Hainsworth et al.^100^	42	FFPE/RNA	CancerTYPE ID (92-gene qRT PCR)	54–86%	Clinicopathological features
Hainsworth et al.*^101^	252	FFPE/RNA	CancerTYPE ID (92-gene qRT PCR)	98%	Clinicopathological features
Greco et al.^102^	149	FFPE/RNA	CancerTYPE ID (92-gene qRT PCR)	70–77	Clinicopathological features
Ades et al.*^103^	67	FFPE/RNA	GEP (microarray) (CUPprint)	82%	Clinicopathological features
Sanden et al.^104^	192	FFPE/microRNA	64 miR-based array	86%	Clinicopathological features
Mileshkin et al.^105^	58	FFPE/RNA	GEP (whole-genome expression)	78%	Conventional histology
Tothill et al.^106^	49	FFPE/RNA	GEP (microarray)	77%	Conventional histology
Moran et al.^107^	216	FFPE/DNA	DNA methylation microarray (EPICUP)	87%	Clinicopathological features/autopsy
Raghav et al.^108^	1834	FFPE/RNA	CancerTYPE ID (92-gene qRT PCR)	94%	Clinicopathological features
Mileshkin et al.*^109^	124	NA/RNA and DNA	386-gene CCP/CUPguide	86.6%	Clinicopathological features

*N* number of patients with CUP, *FF* fresh frozen, *FFPE* formalin fixed paraffin embedded, *NA* not available.

All studies are retrospective except those marked with *.

## The aetiology of CUP

The evidence for germline susceptibility to CUP is weak, and cannot be confirmed by familial studies as the occurrence of cancers in relatives might be incidental and not related to genetic susceptibility.^23^ However, familial studies are informative of both genetic and environmental factors and might be informative of the CUP pathogenesis.^24^ Patients with CUP show significant associations between the location of their CUP and the primary cancer sites in first-degree relatives.^25^ Moreover, many of these patients die of CUP at the same metastatic sites as the primary cancers diagnosed in their relatives.^26^ These findings favour the presence of common genetic mechanisms between certain primary cancers and CUP.^26^ Environmental factors show that smoking increases the risk of CUP, whereas alcohol consumption and the level of education have weaker associations that are attenuated and no longer statistically significant after adjusting for smoking and indices of obesity.^27^ Human papillomavirus (HPV) has been associated with some forms of squamous cell CUP of the head and neck, as well as abdomen, pelvis and retroperitoneum.^28,29^

## The pathogenesis of CUP

**Fig. 1:**
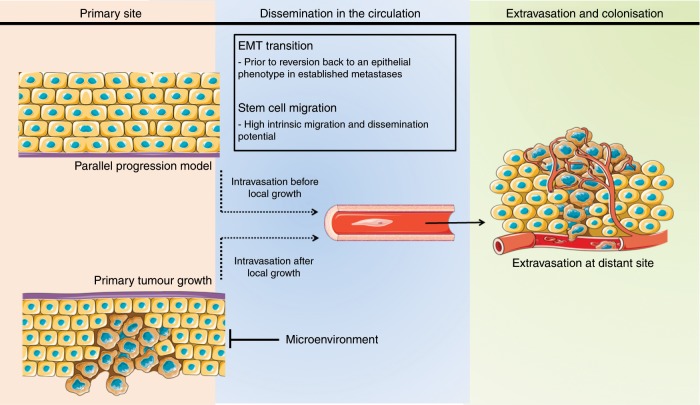
**The successive steps in the carcinogenesis of CUP.**

According to current understanding, the process of tumorigenesis involves the consecutive sequence of clonal proliferation, invasion and intravasation of cancer cells from the primary tumour, dissemination through the circulation, extravasation in different organs and colonisation at metastatic sites (Fig. 1).^30^

Clonal proliferation arises directly from normal stem cells or non-stem cells acquiring DNA alterations that result in the activation of stem-cell programmes according to a type 2 progression, which assumes the acquisition of a malignant phenotype directly without developing premalignant lesions.^31^ Some of these cells are stationary, leading to local tumour growth, whereas others are mobile, yielding distant metastasis.^32^ In the context of CUP, metastasis might occur before local tumour growth as a consequence of two scenarios (Fig. 1). In the first scenario, mobile cells spread at an early stage to metastatic sites and alter their microenvironment, leading to metastasis before the generation of a detectable primary tumour or even transformation into a malignant stage at the primary site.^31,33,34^ This theory is supported by the parallel progression model, with tumour cells showing independent genetic alterations at the primary tumour site and metastatic sites.^35^ In the second scenario, metastasis occurs without parallel progression, with the tumour microenvironment selectively favouring the outgrowth of tumour cells at the metastatic site, while it abrogates the growth of these genetically identical cells at the primary site.^36,37^ This hypothesis is supported by the existence of a clonal relationship between cells at the primary and metastatic sites.^38^

Tumour cells are widely hypothesised to emigrate from their primary site by epithelial-to-mesenchymal transition (EMT).^39^ EMT is a complex process by which mobile cells lose their epithelial phenotype by downregulating E-cadherin, thereby enabling detachment from neighbouring epithelial cells (which continue to display cell–cell adhesion, polarity and lack of motility), and acquire mesenchymal features by upregulating metalloproteinases, which facilitate navigation through the local extracellular matrix and intravasation (motility, invasiveness and increased resistance to apoptosis) to enter the bloodstream or lymphatic system.^39^ Once in the circulation, mobile cells must survive shear and immunological pressures, exit from circulation by extravasation and successfully seed within the metastatic sites.^39^ The EMT phenotype, defined by partial loss of E-cadherin expression with simultaneous expression of N-cadherin/vimentin along with concomitant expression of SNAIL, as assessed by the percentage of staining tumour cells, has been described in 7.3–16% of patients with CUP; this low value probably reflects the transient nature of the phenomenon, which occurs during dissemination prior to reversion back to an epithelial phenotype in established metastases.^40,41^ Nevertheless, the EMT phenotype is associated with high histological grade, presence of visceral metastases and poor survival.^40^ Another hypothesis attributes the tumour expansion to cancer stem cells, which are characterised by a high intrinsic migration/dissemination potential.^42^ The stem cell phenotype, defined by the immunohistochemical expression of CD133 and octamer-binding transcription factor 4 (OCT4), contributes to rapid metastasis in CUP. However, CUP-circulating tumour cells do not express aldehyde dehydrogenase-1, which underlines possibly a transient or rare event.^43^

## Potential hallmarks of CUP

The process that gives rise to CUP, characterised by early metastatic spread, regression of the primary site and aggressive course of the disease, is driven by multiple interdependent alterations in cell behaviour, including chromosomal alterations, self-sufficiency in growth signals, resistance to growth-inhibitory signals, reprogramming of energy metabolism, evasion of apoptosis, limitless replicative potential, sustained angiogenesis, tissue invasion and metastasis and evasion of immune destruction.^44^ The predominant alterations in the carcinogenic pathways have direct implications on the final morphology and natural history of CUP.^45^ For example, adenocarcinoma has the largest number of cell-signalling pathway variants (*EGFR* [epithelial growth factor receptor], *MET*, *JAK3* [Janus kinase 3], *KRAS*, *HRAS*, *BRAF*, *PIK3CA* [phosphatidylinositol-4, 5-bisphosphate 3-kinase catalytic subunit α], *PTPN11* [protein tyrosine phosphatase non-receptor type 11] and *APC*), whereas squamous cell carcinoma shows a higher number of variants in cell-cycle regulation genes (*TP53* [tumour protein p53] and *CDKN2A* [cyclin-dependent kinase inhibitor 2A]). There are no associations with other clinicopathological parameters, such as age, gender or anatomical site of presentation.^45^

**Fig. 2:**
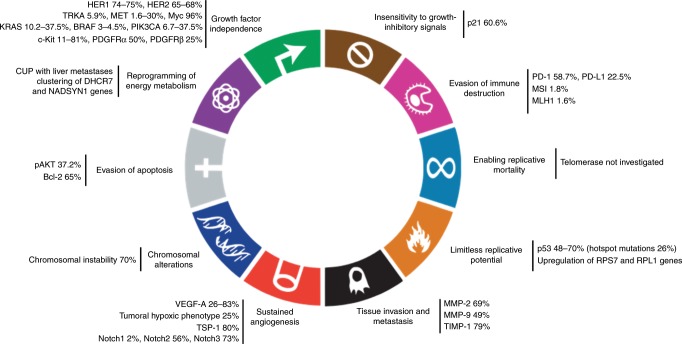
**Putative biological hallmarks in the pathogenesis of CUP.**

In the subsequent sections we describe the hallmarks of cancer (Fig. 2) and their clinical implications in order to rationalise the complexities of the CUP carcinogenesis and difficulties in management.

### Chromosomal alterations

Tumorigenesis requires tens of thousands of genetic alterations that cannot be induced by the normal rate of mutation and therefore requires some form of intrinsic genomic instability to create a mutator phenotype.^46^ Chromosomal instability (CIN) is a process that accelerates the rate of gains or losses of whole or large portions of chromosomes.^47^ Around 70% of patients with CUP show an enrichment in transcripts for proteins that function in DNA damage and homologous recombination repair networks, such as *BRCA1*, *ATM* and *CHEK2* [checkpoint kinase 2], suggesting that CUP is chromosomally unstable.^48^ The most widely accepted molecular basis for CIN involves the oncogene-induced collapse of DNA replication forks, leading to double-stranded DNA breaks and genomic instability.^49^ The consequences of CIN are aneuploidy, subchromosomal genomic amplifications and a high frequency of loss of heterozygosity, resulting in karyotypic variability from cell to cell, which promotes the accumulation of transforming genotypes and increases the acquisition of atypical phenotypes.^48^ Thus, CIN reconciles many of the characteristics of CUP, including poor outcome and drug resistance. Moreover, CIN has also been linked to inflammation by promoting the cGAS–STING (cGMP-AMP synthase-stimulator of interferon genes) pathway (an inflammatory response to the presence of cytosolic DNA), and to the immune system by inducing intratumour heterogeneity that activates cytotoxic immunity by increasing tumour immunogenicity. The influence of CIN in promoting metastasis is not yet fully determined.^50^

Analysis of the cytogenetic profile of CUP has also identified other chromosome modifications that might be encountered, such as 1q21, 3p13, 6q15-23, 7q22, 11p12-5 and 11q14-24.^51^ Adenocarcinomas have a preferential involvement of 4q31, 6q15, 10q25 and 13q22, whereas other carcinomas have a 11q22 involvement. It is noteworthy that massive chromosome changes are associated with poor prognosis.^51^

### Reprogramming of energy metabolism

Altered energy metabolism seems to be involved in carcinogenesis, as germline genetic analysis shows that perturbed lipid metabolism increases the risk of CUP.^23^ CUP with metastases in the liver shows genome-wide associations for chromosome 11 region clustering genes, mainly *DHCR7* (7-dehydrocholesterol reductase) and *NADSYN1* (NAD synthetase 1), which encode two key enzymes involved in cholesterol and NAD synthesis.^23^

### Growth factor independence

The prognostic potential of the activation of two mitogenic signalling pathways, wingless-type (Wnt) and hedgehog (HH), has been studied in CUP.^52^ The downstream Wnt effectors TCF and LEF, as well as the HH pathway effectors SMO and GL1, are expressed in a minority of CUP.^52^ The activation of Wnt pathway is a positive prognostic factor, whereas no prognostic significance of the HH pathway activation has been identified.^52^

Several somatic mutations in growth factor receptors that are predicted to confer constitutive activation of signalling circuits have been identified.^11^ The expression of HER1, also known as EGFR, is reported in 74–75% (overexpression in 4–61%) and HER2 in 65–68% (overexpression in 4–27%) of patients with CUP.^53–56^ The expression of HER1 is not associated with the clinical characteristics of patients with CUP but correlates with sensitivity to platinum-based regimens. Overexpressors of HER2 have predominant supradiaphragmatic involvement and an increased rate of poorly differentiated adenocarcinoma. Its expression is not associated with platinum sensitivity. Few CUP cases with EGFR alterations (not detailed in the corresponding papers) may be successfully managed with anti-EGFR agents.^54,57,58^ The expression of tropomyosin-related kinase A (TRKA), encoded by *NTRK1*, which is known to have oncogenic activity, has been reported in 5.9% of patients with CUP.^59^

KRAS belongs to the *RAS* gene family, members of which relay signals of proliferation, migration, angiogenesis and apoptosis inhibition in the RAS/RAF/mitogen-activated protein kinase (MAPK) kinase (MEK)/extracellular signal-regulated kinase (ERK) (known also as MAPK) intracellular pathway to the downstream effectors BRAF, MEK and PIK3CA.^60^ The expression of RAS is reported in 92% (overexpression 23%) of patients with CUP.^61^ Activating *KRAS* mutations occur in 10.2–37.5%,^62–64^ *BRAF* in 3–4.5%^62,63^ and *PIK3CA* in 6.7–37.5% of patients.^62–64^ Overall, RAS-pathway-activating mutations are described in 15% of patients with CUP, but no single or complex gene mutational profile of this pathway has shown prognostic significance.^62,63^ The expression of MAPK is described in 54% of patients with CUP and is associated with a positive predictive value for response to chemotherapy.^65^ Circulating tumour DNA revealed alterations in the MAPK pathway in 31.2% of patients and in phosphoinositide 3-kinase (PI3K) signalling in 18.1% of patients.^66^

Activation of the *MET* gene, which is known to mediate invasive growth, commonly occurs as a late event in solid tumours and is associated with increased metastatic potential. Nevertheless, it is possible that MET activation constitutes an early event in CUP, resulting in early, systemic dissemination of the malignancy.^67^ Genetic analysis has identified *MET* mutations in 1.6–30% of patients with CUP, which exceeds the 4% prevalence in other tumours.^63,68^ The mutations detected affect the tyrosine kinase domain (p.Val1312Ile) and the extracellular SEMA domain (p.Gln142X; p.His150Tyr; p.Glu168Asp; p.Cys385Tyr); the p.Val1312Ile variant is tumorigenic, whereas the p.Gln142X, p.His150Tyr, p.Glu168Asp and p.Cys385Tyr variants are found in haematogenous metastases to the brain.^68,69^ The expression of cMET is prevalent in the squamous cervical and inguinal nodal subgroups (100% positive for cMET) and is associated with fewer metastatic sites and low-grade squamous tumours.^65^ These findings underline a favourable prognostic impact of cMET in patients with CUP, whereas cMET is actually hypothesised to activate the metastatic potential of CUP.^65,70^ This discrepancy could be explained by a neoplasm- and/or microenvironment-dependent modulation of cMET action.^65^

Other oncogenes that are less well studied include *c-Kit* and *c-Myc*. The oncogene *c-Kit*, known to encode a type III transmembrane receptor with cytoplasmic tyrosine kinase activity, is involved in inhibition of apoptosis, regulation of cell adhesion and induction of cellular proliferation. The expression of c-Kit is reported in 11–81% (overexpression in 4–13%) of patients with CUP.^53–56^ The platelet-derived growth factor receptor (PDGFR), which is known to have cell growth regulation activity, has two subunits, PDGFRα and β, which are expressed in 50% and 25% of patients with CUP, respectively.^71,72^ However, the expression of KIT or PDGFRα did not demonstrate any prognostic utility for response to therapy or survival.^55,58^ The oncogene *Myc* is also involved in the induction of cell proliferation and growth. In patients with CUP, c-Myc expression is reported in 96% (overexpression in 23%)^61^ and MYC amplification in 12% of cases.^45^ Unfortunately, the clinical implications of MYC have not been assessed in the literature reporting on CUP.

### Insensitivity to growth-inhibitory signals

Growth-inhibitory signals can block the proliferation of tumour cells via multiple mechanisms. Alteration in the pathway mediated by the tumour-suppressor protein retinoblastoma (Rb) renders tumours insensitive to growth-inhibitory factors that block transition through the G1 phase of the cell cycle. Phosphorylation of Rb, a process that facilitates entry into the cell cycle, is induced by cyclin–CDK complexes; these cyclin–CDK complexes, in turn, are inhibited by p21 proteins. The high expression of p21 proteins is reported in 60.6% of patients with CUP and is associated with better survival.^70,73^ The expression levels of p21 correlate with different CUP subgroups, with high p21 expression seen predominantly in nodal CUP (76% versus 63% in visceral CUP versus 44% of the peritoneal or pleural carcinomatosis; *P* = 0.025).^73^

### Evasion of apoptosis

The ability of tumours to grow is determined not only by the rate of cell proliferation but also by the rate of cell attrition. Moreover, phosphatase and tensin homologue (PTEN), a tumour suppressor that normally attenuates the AKT survival signal, is reported in 50% of CUP.^73^ The AKT pathway, which confers anti-apoptotic signals, is probably involved in preventing apoptosis in CUP. The expression of phosphorylated AKT (pAKT) is reported in 73.2% of patients with CUP, and is associated with worse survival.^70,73^

The expression of the anti-apoptotic protein Bcl-2 is reported in 65% (overexpression in 40%) of patients with CUP.^74^ This finding is unexpected, as Bcl-2 is commonly upregulated in premalignant lesions rather than in advanced malignancies, and is usually associated with a less aggressive phenotype.^75^ The expression of Bcl-2 has no prognostic value in CUP, but its co-expression with p53 might be correlated with a more favourable response to cisplatin-based chemotherapy.^74^

### Limitless replicative potential

The expression of p53 is reported in 48–70% (overexpression in 53%) of patients with CUP, although it has no prognostic value for treatment benefit or survival.^45,56,74,76^ p53 hotspot mutations, which reside within exons 5–9, are reported in 26% of patients with CUP.^77^ The discordance between the expression and mutational analysis data could be attributed to the differing specificities of antibodies for wild-type and mutated p53 gene products, as well as the occurrence of mutations outside of the exon 5–9 region.^77^ Alterations in *TP53*-associated genes, such as *TP53* and *ATM* substitution, have been reported in 37.8% of patients with CUP following circulating tumour DNA analysis; however, the clinical implications in patients with CUP have not been reported.^66^ The functions of p53 are regulated by the oncoprotein MDM2, which is encoded by a p53 transcriptional target gene.^78^ *RPS7* (ribosomal protein S7) and *RPL1* (ribosomal protein L1), two genes that encode ribosomal proteins involved in the MDM2/p53 pathway, are shown to be upregulated in patients with CUP. These genes increase the potential of EMT that favours metastatisation.^78^

### Sustained angiogenesis

The progression of tumorigenesis usually involves the activation of an ‘angiogenic switch’, which is required for the growth of a lesion beyond a certain size. It is hypothesised that CUP presents an angiogenic incompetence at the primary site, which thereby limits the development of the primary tumour.^79^ Metastatic sites might overcome this angiogenic incompetence either because the new tumour microenvironment supports growth of tumour clones without angiogenesis or because of the occurrence of additional genetic alterations during metastasis.^80,81^ Although metastatic sites of CUP show a high degree of vascularisation, the available data do not show a specific biological role for angiogenesis in the metastatic phenotype of CUP.^82^

Vascular endothelial growth factor-A (VEGF-A), expressed in 26–83% of patients with CUP, is a well-known signalling protein that binds to stimulatory cell-surface receptors expressed by vascular endothelial cells.^57,76,83,84^ Its expression does not have any prognostic implications, although previously published data showed that tumour microvessel density was an adverse prognostic factor in patients with liver metastases of unknown primary.^82^ VEGF can be sequestered in the extracellular matrix in latent forms and later released and activated by extracellular matrix-degrading proteases. Its expression can be upregulated both by hypoxia and by oncogene signalling. The tumoural hypoxic phenotype, defined by the expression of hypoxia-inducible factor 1a (HIF1a), glucose transporter 1 (GLUT1) and cyclo-oxygenase 2 (COX2), is described in 25% of patients with CUP, and has been reported to be associated with a worse prognosis.^72^ The angiogenesis regulator gene *TXR1* and hypoxic factor *HIFa* are significantly associated with a decreased survival in patients with CUP (7.4 versus 18.3 months for low versus high *TXR1* mRNA expression and 6.9 versus 19.8 months for low versus higher *HIF1a* mRNA expression).^85^

In addition to VEGF, multiple ligands that are functionally implicated in developmental and tumour-associated angiogenesis have been evaluated in patients with CUP. Thrombospondin-1 (TSP-1), expressed in 80% of patients with CUP (overexpression in 20%), binds transmembrane receptors expressed by endothelial cells and induces suppressive signals that counteract proangiogenic stimuli.^83^ Its expression is not associated with any clinical or pathological parameters.^83^ The different isoforms of Notch are expressed at different levels in patients with CUP: 2%, 56% and 73% for Notch1, Notch2 and Notch3, respectively, whereas the expression level of Notch4 was not reported.^65^ Notch1 and Notch2 share the highest homology, whereas the Notch3 receptor lacks the transactivation domain. No clarity currently exists regarding specificity in Notch signalling with respect to each Notch protein. However, the overexpression of Notch3 correlates with worse survival in the subset of patients with midline nodal CUP, while the overexpression of Notch1 is linked to inferior survival in visceral CUP.^65^

### Tissue invasion and metastasis

Metalloproteinase (MMP)-2 and MMP-9 are expressed in 69% (overexpression in 49%) and 49% (overexpression in 36%) of patients with CUP, respectively.^84^ These expression levels are decreased by the knockdown of proteasome subunit β type-4 (PSMB4) through deactivating the proteasome cascades.^86^ The expression of MMP-2 and MMP-9 is not associated with tumour differentiation, clinical subgroups, response to treatment or survival.^84^ Metastasis-suppressor genes are genes that decrease the capability of the malignant clone to degrade the surrounding extracellular matrix. The expression of tissue inhibitor of metalloproteinases (TIMP-1) is reported in 79% of patients with CUP, and is associated with a shorter survival.^84^ The loss of function of the metastasis suppressor *KiSS-1* is seen in several human malignancies and is correlated with advanced stage and poor prognosis. However, such a loss of function is extremely rare in patients with CUP, although this does not rule out epigenetic suppression of gene transcription or post-translational protein modifications.^87^

### Evasion of immune destruction

As part of the process of tumorigenesis, cancer cells inhibit the immune system partly by targeting the inhibitory pathway of programmed cell death protein (PD-1) and its ligand (PD-L1). PD-1 expression was detected in the tumour-infiltrating lymphocytes of 58.7% of patients with CUP, whereas PD-L1 expression was seen in 22.5% of cancer cells in tumours, respectively.^56^ In the absence of an accurate predictive biomarker for immune checkpoint inhibitors, the expression of PD-(L)1 is the rational biomarker for PD-L(1) inhibitors. The available literature in CUP is limited to case reports of patients treated with immune checkpoint inhibitors that limit solid conclusions.^9^

Other markers of the immune microenvironment include the tumour mutation load, which is reported as being ‘high’ in 11.8% of patients, and microsatellite instability, which is ‘high’ in 1.8% of patients with CUP.^56^ Mutations in MLH1 (mutL homologue 1), a DNA mismatch repair protein, have been detected in 1.6% of patients with CUP using circulating tumour DNA.^66^ MSI (microsatellite instability) is associated with a high tumour mutational burden and is a predictive biomarker of response to immune checkpoint inhibitors in a multitude of tumours. Pembrolizumab, an anti-PD-1 agent, is approved in patients with unresectable or metastatic tumours with mismatch-repair deficiency.^88^

Macrophage migration-inhibitory (MIF) factor is an important regulator of innate immunity that has been implicated in CUP pathogenesis as its knockdown suppresses metastasis.^86^ Its overexpression has prognostic significance associated with a shorter survival. The poor clinical outcome is explained by the potentiating effects on angiogenesis (by activating HIF1a), proliferation (by stimulating MAPK pathway and inhibiting p53) and invasion (partly via MMP-9).^89^

Leukotriene A4 hydrolase (LTA4H) catalyses the final step in biosynthesis of leukotriene B4, a strong chemotactic factor for mast cells and neutrophils that has been implicated in the inflammation-driven development of CUP by increasing the transcription of oncogenes and interfering with apoptosis.^23^

## Conclusion

We have sought here to extend the concept of cancer hallmarks to CUP in order to provide a useful conceptual framework for understanding the enigmatic biology of CUP. This conceptualisation allows us to understand the acquired functional capabilities that allow the putative cells to survive, proliferate and disseminate (Figs. 1 and 2). Further refinement of these organising principles is foreseeable in the near future as our analysis of the genetic and molecular profiling is advancing. Indeed, the use of profiling platforms in the clinical management of patients with cancer, and particularly in CUP, is now a reality. Moreover, the success of targeted therapy in several solid tumours has boosted the interest in patients with CUP, and although the application of molecular diagnostics has indeed facilitated study of the molecular pathogenesis and biological behaviour in CUP, it still lacks survival benefit in clinical practice.^10–12^

Currently, of all the putative hallmarks for CUP, the acquisition of chromosomal instability has received most of the attention in promoting the accumulation of transforming genotypes and increasing the acquisition of independent phenotypic traits. Current research focuses mainly on growth factor independence, and little research has been performed on the remaining hallmarks, despite their important role in promoting and regulating the carcinogenesis of CUP. Further investigation of these hallmarks might well help not only to uncover the enigmatic features of CUP, but also to identify new, ‘actionable’ driver targets for its treatment. The improved knowledge of the biology of CUP has identified particular subsets such as colorectal, lung and renal CUP profiles that provide guidance in the tailoring of specific therapies. The novel profiling techniques are expected to bridge the gaps in the understanding of the CUP tumorigenesis. We anticipate an overall holistic clarity of the different hallmarks that elicit the clinical implications of the cellular interplay in CUP instead of just reporting the occurrence of some pathways. This approach would allow a better understanding of the CUP biology and eventually designing more effective treatments.

## Data Availability

Not applicable.

## References

[1] Fizazi K, Greco FA, Pavlidis N, Daugaard G, Oien K, Pentheroudakis G (2015). Cancers of unknown primary site: ESMO Clinical Practice Guidelines for diagnosis, treatment and follow-up. Ann. Oncol. J. Eur. Soc. Med Oncol..

[2] Pavlidis N, Pentheroudakis G (2012). Cancer of unknown primary site. Lancet Lond. Engl..

[3] Kim CS, Hannouf MB, Sarma S, Rodrigues GB, Rogan PK, Mahmud SM (2018). Survival outcome differences based on treatments used and knowledge of the primary tumour site for patients with cancer of unknown and known primary in Ontario. Curr. Oncol..

[4] Pentheroudakis G. Pavlidis N. Serum tumor markers. In Wick M. R., ed. Metastatic carcinomas of unknown origin. New York, NY: Demos Medical Publishing, 2008: 165–175. n.d.

[5] Delgado-Bolton RC, Fernández-Pérez C, González-Maté A, Carreras JL (2003). Meta-analysis of the performance of 18F-FDG PET in primary tumor detection in unknown primary tumors. J. Nucl. Med Publ. Soc. Nucl. Med..

[6] Hainsworth JohnD, Greco FAnthony (2018). “Cancer of Unknown Primary Site: New Treatment Paradigms in the Era of Precision Medicine.”. Am. Soc. Clin. Oncol. Educ. Book.

[7] Rassy EE, Khaled H, Pavlidis N (2018). Liquid biopsy: a new diagnostic, predictive and prognostic window in cancers of unknown primary. Eur. J. Cancer.

[8] Moran S, Martinez-Cardús A, Boussios S, Esteller M (2017). Precision medicine based on epigenomics: the paradigm of carcinoma of unknown primary. Nat. Rev. Clin. Oncol..

[9] Rassy EE, Pavlidis N (2019). The currently declining incidence of cancer of unknown primary. Cancer Epidemiol..

[10] Hayashi H, Kurata T, Takiguchi Y, Arai M, Takeda K, Akiyoshi K (2019). Randomized phase II trial comparing site-specific treatment based on gene expression profiling with carboplatin and paclitaxel for patients with cancer of unknown primary site. J. Clin. Oncol..

[11] Rassy EE, Pavlidis N (2018). The current evidence for a biomarker-based approach in cancer of unknown primary. Cancer Treat..

[12] Fizazi K., Maillard A., Penel N., Baciarello G., Allouache D., Daugaard G., et al. LBA15_PRA phase III trial of empiric chemotherapy with cisplatin and gemcitabine or systemic treatment tailored by molecular gene expression analysis in patients with carcinomas of an unknown primary (CUP) site (GEFCAPI 04). *Ann. Oncol*. **30**, mdz394 (2019). 10.1093/annonc/mdz394.

[13] Pavlidis N (2007). Forty years experience of treating cancer of unknown primary. Acta Oncol..

[14] Bochtler T., Krämer A. Does cancer of unknown primary (CUP) truly exist as a distinct cancer entity? *Front. Oncol*. **9**, 402 (2019) 10.3389/fonc.2019.00402 PMC6534107 31165045

[15] Hainsworth JD, Greco FA (2014). Gene expression profiling in patients with carcinoma of unknown primary site: from translational research to standard of care. Virchows Arch Int. J. Pathol..

[16] Bochtler T, Endris V, Leichsenring J, Reiling A, Neumann O, Volckmar A-L (2019). Comparative genetic profiling aids diagnosis and clinical decision making in challenging cases of CUP syndrome. Int J. Cancer.

[17] Rassy EE, Kattan J, Pavlidis N (2019). Familial cancer of unknown primary. Int J. Clin. Oncol..

[18] Conway, A.-M., Mitchell, C., Kilgour, E., Brady, G., Dive, C. & Cook, N. Molecular characterisation and liquid biomarkers in Carcinoma of Unknown Primary (CUP): taking the ‘U’ out of ‘CUP.’ *Br. J. Cancer* **120**, 141 (2019). 10.1038/s41416-018-0332-2 PMC6342985 30580378

[19] Schreiber RD, Old LJ, Smyth MJ (2011). Cancer immunoediting: integrating immunity’s roles in cancer suppression and promotion. Science.

[20] Rassy EE, Assi T, Kattan J, Pavlidis N (2019). Paraneoplastic syndromes in cancers of unknown primary: an unknown field for oncologists. Bull. Cancer (Paris).

[21] Vikeså, J., Møller, A. K. H., Kaczkowski, B., Borup, R., Winther, O., Henao, R. et al. Cancers of unknown primary origin (CUP) are characterized by chromosomal instability (CIN) compared to metastasis of know origin. *BMC Cancer* **15**, 151 (2015). 10.1186/s12885-015-1128-x PMC4404593 25885340

[22] Pentheroudakis G, Briasoulis E, Pavlidis N (2007). Cancer of Unknown Primary site: missing primary or missing biology?. Oncologist.

[23] Hemminki, K, Chen, B, Kumar, A, Melander, O, Manjer, J., Hallmans, G. et al. Germline genetics of cancer of unknown primary (CUP) and its specific subtypes. *Oncotarget*. **7**, 22140–22149 (2016). 10.18632/oncotarget.7903 PMC5008350 26959888

[24] Hemminki K, Ji J, Sundquist J, Shu X (2011). Familial risks in cancer of unknown primary: tracking the primary sites. J. Clin. Oncol..

[25] Hemminki K., Sundquist K., Sundquist J., Hemminki A., Ji J. Location of metastases in cancer of unknown primary are not random and signal familial clustering. *Sci. Rep*. 22891, **6** (2016). 10.1038/srep22891 PMC4783693 26956545

[26] Hemminki, K., Bevier, M., Sundquist, J. & Hemminki, A. Cancer of unknown primary (CUP): does cause of death and family history implicate hidden phenotypically changed primaries? *Ann. Oncol*. **23**, 2720–2724 (2012). 10.1093/annonc/mds063 22473595

[27] Kaaks R, Sookthai D, Hemminki K, Krämer A, Boeing H, Wirfält E (2014). Risk factors for cancers of unknown primary site: results from the prospective EPIC cohort. Int J. Cancer.

[28] Rassy E. E., Kattan J., Pavlidis N. A new entity of abdominal squamous cell carcinoma of unknown primary. *Eur. J. Clin. Invest*. **49**, e13111 (2019). 10.1111/eci.13111 30908618

[29] Rassy E.E., Nicolai P., Pavlidis N. (2019). Comprehensive management of HPV-related squamous cell carcinoma of the head and neck of unknown primary. Head Neck.

[30] Vanharanta S, Massagué J (2013). Origins of metastatic traits. Cancer Cell.

[31] López-Lázaro M (2015). The migration ability of stem cells can explain the existence of cancer of unknown primary site Rethinking metastasis. Oncoscience.

[32] Brabletz T, Jung A, Spaderna S, Hlubek F, Kirchner T (2005). Opinion: migrating cancer stem cells—an integrated concept of malignant tumour progression. Nat. Rev. Cancer.

[33] Califano J, Westra WH, Koch W, Meininger G, Reed A, Yip L (1999). Unknown primary head and neck squamous cell carcinoma: molecular identification of the site of origin. J. Natl Cancer Inst..

[34] Scadden DT (2006). The stem-cell niche as an entity of action. Nature.

[35] Joosse SA, Pantel K (2016). Genetic traits for hematogeneous tumor cell dissemination in cancer patients. Cancer Metastasis Rev..

[36] Suzuki M, Mose ES, Montel V, Tarin D (2006). Dormant cancer cells retrieved from metastasis-free organs regain tumorigenic and metastatic potency. Am. J. Pathol..

[37] Tarin D (2012). Clinical and biological implications of the tumor microenvironment. Cancer Microenviron..

[38] Speel E-JM, van de WouwAJ, SMH Claessen, Haesevoets A, AHN Hopman, van der WurffAAM (2008). Molecular evidence for a clonal relationship between multiple lesions in patients with unknown primary adenocarcinoma. Int J. Cancer.

[39] Dasgupta A, Lim AR, Ghajar CM (2017). Circulating and disseminated tumor cells: harbingers or initiators of metastasis?. Mol. Oncol..

[40] Stoyianni A, Goussia A, Pentheroudakis G, Siozopoulou V, Ioachim E, Krikelis D (2012). Immunohistochemical Study of the Epithelial-Mesenchymal Transition Phenotype in Cancer of Unknown Primary: incidence, correlations and prognostic utility. Anticancer Res..

[41] Stoyianni A, Pentheroudakis G, Benjamin H, Cervantes A, Ashkenazi K, Lazaridis G (2014). Insights into the epithelial mesenchymal transition phenotype in cancer of unknown primary from a global microRNA profiling study. Clin. Transl. Oncol..

[42] Sampieri K, Fodde R (2012). Cancer stem cells and metastasis. Semin Cancer Biol..

[43] Kamposioras K, Pentheroudakis G, Pavlidis N (2013). Exploring the biology of cancer of unknown primary: breakthroughs and drawbacks. Eur. J. Clin. Invest.

[44] Hanahan D, Weinberg RA (2011). Hallmarks of cancer: the next generation. Cell.

[45] Clynick B, Dessauvagie B, Sterrett G, Harvey NT, Allcock RJN, Saunders C (2018). Genetic characterisation of molecular targets in carcinoma of unknown primary. J. Transl. Med..

[46] Loeb LA (2016). Human cancers express a mutator phenotype: hypothesis, origin, and consequences. Cancer Res..

[47] Lengauer C, Kinzler KW, Vogelstein B (1998). Genetic instabilities in human cancers. Nature.

[48] Hedley DW, Leary JA, Kirsten F (1985). Metastatic adenocarcinoma of unknown primary site: abnormalities of cellular DNA content and survival. Eur. J. Cancer Clin. Oncol..

[49] Negrini S, Gorgoulis VG, Halazonetis TD (2010). Genomic instability-an evolving hallmark of cancer. Nat. Rev. Mol. Cell Biol..

[50] Tijhuis AE, Johnson SC, McClelland SE (2019). The emerging links between chromosomal instability (CIN), metastasis, inflammation and tumour immunity. Mol. Cytogenet.

[51] Pantou D, Tsarouha H, Papadopoulou A, Mahaira L, Kyriazoglou I, Apostolikas N (2003). Cytogenetic profile of unknown primary tumors: clues for their pathogenesis and clinical management. Neoplasia N. Y N..

[52] Fotopoulos G, Gousia A, Bareta E, Koumpis E, Chrisafi S, Bobos M (2015). Prognostic significance of WNT and hedgehog pathway activation markers in cancer of unknown primary. Eur. J. Clin. Invest.

[53] Hainsworth JD, Lennington WJ, Greco FA (2000). Overexpression of Her-2 in patients with poorly differentiated carcinoma or poorly differentiated adenocarcinoma of unknown primary site. J. Clin. Oncol..

[54] Massard C, Voigt J-J, Laplanche A, Culine S, Lortholary A, Bugat R (2007). Carcinoma of an unknown primary: are EGF receptor, Her-2/neu, and c-Kit tyrosine kinases potential targets for therapy?. Br. J. Cancer.

[55] Dova L, Pentheroudakis G, Golfinopoulos V, Malamou-Mitsi V, Georgiou I, Vartholomatos G (2008). Targeting c-KIT, PDGFR in cancer of unknown primary: a screening study for molecular markers of benefit. J. Cancer Res Clin. Oncol..

[56] Gatalica Z, Xiu J, Swensen J, Vranic S (2018). Comprehensive analysis of cancers of unknown primary for the biomarkers of response to immune checkpoint blockade therapy. Eur. J. Cancer Oxf. Engl. 1990.

[57] Rashid A, Hess KR, Lenzi R (2005). Overexpression and prevalence of molecular markers in patients with cancer of unknown primary (CUP). Proc. Am. Soc. Clin. Oncol..

[58] Dova L, Georgiou I, Vartholomatos G (2005). EGFR and C-KIT/CD117 gene mutational screening and oncoprotein expression in patients with cancer of unknown primary: Implications for molecular pathophysiology and therapy. Eur. J. Cancer.

[59] Mauri G, Valtorta E, Cerea G, Amatu A, Schirru M, Marrapese G (2018). TRKA expression and NTRK1 gene copy number across solid tumours. J. Clin. Pathol..

[60] Rajalingam K., Schreck R., Rapp U. R., Albert Š. Ras oncogenes and their downstream targets. *Biochim. Biophys. Acta* **1773**, 1177–1195 (2007) 10.1016/j.bbamcr.2007.01.012 17428555

[61] Pavlidis N, Briassoulis E, Bai M, Fountzilas G, Agnantis N (1995). Overexpression of C-myc, Ras and C-erbB-2 oncoproteins in carcinoma of unknown primary origin. Anticancer Res.

[62] Gatalica Z. Molecular profiling of cancers of unknown primary site (CUP): Paradigm shift in management of CUP. Presented at ECCO Cancer Congress 2013: Abstract LBA39 n.d. http://www.ecco-org.eu/Amsterdam2013/Global/News/ECC-2013-Press-Releases-EN/2013/09/Identifying-the-disease-causing-mechanisms-in-cancers.aspx.

[63] Pentheroudakis G, Kotteas EA, Kotoula V, Papadopoulou K, Charalambous E, Cervantes A (2014). Mutational profiling of the RAS, PI3K, MET and b-catenin pathways in cancer of unknown primary: a retrospective study of the Hellenic Cooperative Oncology Group. Clin. Exp. Metastasis.

[64] Tothill RW, Li J, Mileshkin L, Doig K, Siganakis T, Cowin P (2013). Massively-parallel sequencing assists the diagnosis and guided treatment of cancers of unknown primary. J. Pathol..

[65] Krikelis D, Pentheroudakis G, Goussia A, Siozopoulou V, Bobos M, Petrakis D (2012). Profiling immunohistochemical expression of NOTCH1-3, JAGGED1, cMET, and phospho-MAPK in 100 carcinomas of unknown primary. Clin. Exp. Metastasis.

[66] Kato S, Krishnamurthy N, Banks KC, De P, Williams K, Williams C (2017). Utility of genomic analysis in circulating tumor DNA from patients with carcinoma of unknown primary. Cancer Res.

[67] Stella GM, Senetta R, Cassenti A, Ronco M, Cassoni P (2012). Cancers of unknown primary origin: current perspectives and future therapeutic strategies. J. Transl. Med.

[68] Stella GM, Benvenuti S, Gramaglia D, Scarpa A, Tomezzoli A, Cassoni P (2011). MET mutations in cancers of unknown primary origin (CUPs). Hum. Mutat..

[69] Graveel C, Su Y, Koeman J, Wang L-M, Tessarollo L, Fiscella M (2004). Activating Met mutations produce unique tumor profiles in mice with selective duplication of the mutant allele. Proc. Natl Acad. Sci. USA.

[70] Pentheroudakis G, Petrakis D, Goussia A, Siozopoulou V, Bobos M, Fountzilas G (2011). 1450 POSTER immunohistochemical profiling of signalling pathways in Cancer of Unknown Primary (CUP). Eur. J. Cancer.

[71] Dova L, Pentheroudakis G, Georgiou I, Malamou-Mitsi V, Vartholomatos G, Fountzilas G (2007). Global profiling of EGFR gene mutation, amplification, regulation and tissue protein expression in unknown primary carcinomas: to target or not to target?. Clin. Exp. Metastasis.

[72] Koo JS, Kim H (2011). Hypoxia-related protein expression and its clinicopathologic implication in carcinoma of unknown primary. Tumor Biol..

[73] Golfinopoulos, V., Pentheroudakis, G., Goussia, A., Siozopoulou, V., Bobos, M., Krikelis, D. et al. Intracellular signalling via the AKT axis and downstream effectors is active and prognostically significant in cancer of unknown primary (CUP): a study of 100 CUP cases. *Ann. Oncol*. **23**, 2725–2730 (2012). 10.1093/annonc/mds097 22565124

[74] Briasoulis E, Tsokos M, Fountzilas G, Bafaloukos D, Kosmidis P, Samantas E (1998). Bcl2 and p53 protein expression in metastatic carcinoma of unknown primary origin: biological and clinical implications. A Hellenic Co-operative Oncology Group study. Anticancer Res..

[75] van de Wouw AJ, Jansen RLH, Speel EJM, Hillen HFP (2003). The unknown biology of the unknown primary tumour: a literature review. Ann. Oncol..

[76] van de Wouw AJ, Jansen RLH, Griffioen AW, Hillen HFP (2004). Clinical and immunohistochemical analysis of patients with unknown primary tumour. A search for prognostic factors in UPT. Anticancer Res..

[77] Bar-Eli M, Abbruzzese JL, Lee-Jackson D, Frost P (1993). p53 gene mutation spectrum in human unknown primary tumors. Anticancer Res..

[78] Fujita Y, Kurahashi I, Kurata T, Koh Y, Sakai K, Nakagawa K (2014). Abstract 3993: A microarray-based gene expression analysis identified diagnostic biomarkers for unknown primary cancer. Cancer Res..

[79] Naresh KN (2002). Do metastatic tumours from an unknown primary reflect angiogenic incompetence of the tumour at the primary site?-a hypothesis. Med. Hypotheses.

[80] Almog N, Ma L, Raychowdhury R, Schwager C, Erber R, Short S (2009). Transcriptional switch of dormant tumors to fast-growing angiogenic phenotype. Cancer Res..

[81] Naumov GN, Bender E, Zurakowski D, Kang S-Y, Sampson D, Flynn E (2006). A model of human tumor dormancy: an angiogenic switch from the nonangiogenic phenotype. J. Natl Cancer Inst..

[82] Hillen HF, Hak LE, Joosten-Achjanie SR, Arends JW (1997). Microvessel density in unknown primary tumors. Int J. Cancer.

[83] Karavasilis V, Malamou-Mitsi V, Briasoulis E, Tsanou E, Kitsou E, Kalofonos H (2005). Angiogenesis in cancer of unknown primary: clinicopathological study of CD34, VEGF and TSP-1. BMC Cancer.

[84] Karavasilis V, Malamou-Mitsi V, Briasoulis E, Tsanou E, Kitsou E, Kalofonos H (2005). Matrix metalloproteinases in carcinoma of unknown primary. Cancer.

[85] Souglakos J., Pentheroudakis G., Papadaki C., Cervantes A., Petrakis D. et al. Prognostic significance of gene expression profile in patients with carcinomas of unknown primary. *Ann. Oncol*. **21**(Suppl. 8), 55 (2010).

[86] Fujita Y, Sakai K, Velasco MD, Kurata T, Hayashi H, Nakagawa K (2018). Abstract 5169: novel target molecules for treatment of cancer of unknown primary. Cancer Res.

[87] Dova L, Golfinopoulos V, Pentheroudakis G, Georgiou I, Pavlidis N (2008). Systemic dissemination in cancer of unknown primary is independent of mutational inactivation of the KiSS-1 metastasis-suppressor gene. Pathol. Oncol. Res..

[88] Le DT, Uram JN, Wang H, Bartlett BR, Kemberling H, Eyring AD (2015). PD-1 blockade in tumors with mismatch-repair deficiency. N. Engl. J. Med.

[89] Kindt N, Journe F, Laurent G, Saussez S (2016). Involvement of macrophage migration inhibitory factor in cancer and novel therapeutic targets. Oncol. Lett..

[90] Tothill RW, Kowalczyk A, Rischin D, Bousioutas A, Haviv I, Laar RKvan (2005). An expression-based site of origin diagnostic method designed for clinical application to cancer of unknown origin. Cancer Res.

[91] Horlings HM, van Laar RK, Kerst J-M, Helgason HH, Wesseling J, van der Hoeven JJM (2008). Gene expression profiling to identify the histogenetic origin of metastatic adenocarcinomas of unknown primary. J. Clin. Oncol. J. Am. Soc. Clin. Oncol..

[92] Bridgewater J, van Laar R, Floore A, Van’T Veer L (2008). Gene expression profiling may improve diagnosis in patients with carcinoma of unknown primary. Br. J. Cancer.

[93] Varadhachary GR, Talantov D, Raber MN, Meng C, Hess KR, Jatkoe T (2008). Molecular profiling of carcinoma of unknown primary and correlation with clinical evaluation. J. Clin. Oncol..

[94] van Laar RK, Ma X-J, de Jong D, Wehkamp D, Floore AN, Warmoes MO (2009). Implementation of a novel microarray-based diagnostic test for cancer of unknown primary. Int J. Cancer.

[95] Monzon FA, Medeiros F, Lyons-Weiler M, Henner WD (2010). Identification of tissue of origin in carcinoma of unknown primary with a microarray-based gene expression test. Diagn. Pathol..

[96] Ferracin M, Pedriali M, Veronese A, Zagatti B, Gafà R, Magri E (2011). MicroRNA profiling for the identification of cancers with unknown primary tissue-of-origin. J. Pathol..

[97] Varadhachary GR, Spector Y, Abbruzzese JL, Rosenwald S, Wang H, Aharonov R (2011). Prospective gene signature study using microRNA to identify the tissue of origin in patients with carcinoma of unknown primary. Clin. Cancer Res.

[98] Thompson DS, Hainsworth JD, Lane CM, Lennington WJ, Spigel DR, Greco FA (2011). Molecular tumor profiling (MTP) in cancer of unknown primary site (CUP): a complement to standard pathologic diagnosis. J. Clin. Oncol..

[99] Fernandez AF, Assenov Y, Martin-Subero JI, Balint B, Siebert R, Taniguchi H (2012). A DNA methylation fingerprint of 1628 human samples. Genome Res.

[100] Hainsworth JD, Schnabel CA, Erlander MG, Haines DW, Greco FA (2012). A retrospective study of treatment outcomes in patients with carcinoma of unknown primary site and a colorectal cancer molecular profile. Clin. Colorectal Cancer.

[101] Hainsworth JD, Rubin MS, Spigel DR, Boccia RV, Raby S, Quinn R (2013). Molecular gene expression profiling to predict the tissue of origin and direct site-specific therapy in patients with carcinoma of unknown primary site: a prospective trial of the Sarah Cannon research institute. J. Clin. Oncol..

[102] Greco FA, Lennington WJ, Spigel DR, Hainsworth JD (2013). Molecular profiling diagnosis in unknown primary cancer: accuracy and ability to complement standard pathology. J. Natl Cancer Inst..

[103] Ades F, de Azambuja E, Daugaard G, Ameye L, Moulin C, Paesmans M (2013). Comparison of a gene expression profiling strategy to standard clinical work-up for determination of tumour origin in cancer of unknown primary (CUP). J. Chemother. Florence Italy.

[104] Sanden, M. O., Wassman, R., Ashkenazi, K., Benjamin, H., Spector, Y. & Goren, E. Observational study of real world clinical performance of microrna molecular profiling for cancer of unknown primary (CUP). *J. Clin. Oncol*. **31**, e22173–e22173 (2013).

[105] Mileshkin, L. R., Byron, K., Tothill, R., Shi, F., Paiman, L., Bedo, J. et al. Development of a histology-guided gene expression tumor classifier for cancer of unknown primary (CUP). *J. Clin. Oncol*. **32**, 11108–11108 (2014).

[106] Tothill RW, Shi F, Paiman L, Bedo J, Kowalczyk A, Mileshkin L (2015). Development and validation of a gene expression tumour classifier for cancer of unknown primary. Pathol. (Philos.).

[107] Moran S, Martínez-Cardús A, Sayols S, Musulén E, Balañá C, Estival-Gonzalez A (2016). Epigenetic profiling to classify cancer of unknown primary: a multicentre, retrospective analysis. Lancet Oncol..

[108] Raghav, K. P. S., Poage, G. M., Schnabel, C. A. & Varadhachary, G. R. Resolving diagnostic uncertainty in bone-predominant metastases in cancer of unknown primary (CUP) using the 92-gene assay. *J. Clin. Oncol*. **36**, 12064–12064 (2018).

[109] Mileshkin LR, Sivakumaran T, Etemadmoghadam D, Tothill R, Fellowes A, Fox SB (2019). Clinical impact of tissue of origin testing and mutation profiling in the Solving Unknown Primary Cancer (SUPER) national prospective study: Experience of the first two years. J. Clin. Oncol..

